# A case report of perioperative managements for a patient with gastric cancer and cold agglutinin syndrome

**DOI:** 10.1097/MD.0000000000006082

**Published:** 2017-03-10

**Authors:** Ning Xu, Shuli Guo, Jianchun Yu, Yufen Ma

**Affiliations:** Peking Union Medical College Hospital, Beijing, People's Republic of China.

**Keywords:** cold agglutinin disease, gastric cancer, hemolytic anemia, perioperative management

## Abstract

**Rationale:**

Gastric cancer patient with cold agglutinin syndrome (CAS) is an extremely rare entity. This kind of patients is very sensitive to the environment, and they always need scrupulous perioperative treatment, however the experience of perioperative treatment for these patients has been seldom reported.

**Patient concerns:**

The patient was a 54-year-old male. He suffered diarrhea for 3 months, and later the gastroscopy found a tumor located in the gastric antrum and the biopsy was performed. The pathological result reported that it was poorly differentiated gastric adenocarcinoma. The patient was previously diagnosed with CAS 8 years ago.

**Diagnoses:**

Gastric cancer patient with cold agglutinin syndrome (CAS).

**Interventions:**

After 2-month neoadjuvant chemoradiotherapy, the patient underwent open radical distal gastrectomy and D2 lymph node resection. No blood transfusion was performed. Eight days after operation, the patient was discharged. During the perioperative period, scrupulous plan was performed, including careful vital signs monitoring, rigid environment thermal control, infusion warming, proper methods for blood sampling and transmission, and mental relief.

**Outcomes:**

Curative resection was achieved and the patient was discharged. The perioperative period was uneventful.

**Lessons:**

Because of the fragility of CAS, the perioperative management was vital for this patient. Scrupulous plan may guarantee the safety of the patients.

## 1 Introduction

Cold agglutinin syndrome (CAS) is a group of syndromes characterized by chronic hemolytic anemia and microembolism caused by autoreactive hemagglutination and cold-induced factors.^[1]^ Cold agglutinin antibodies are mainly immunoglobulin M (IgM), which can act on their own red cell antigens and lead to reversible hemagglutination when the environmental temperature is <21°C. Patients with cold agglutinin disease may have symptoms related to red cell agglutination when exposed to a cold surrounding temperature. The most common manifestations are livedo reticularis and acrocyanosis. These skin changes could disappear completely within several minutes once the temperature is >21°C.^[2]^ Under some circumstances, the exacerbations of CAS could lead to acute hemolysis, jaundice, and even acute renal dysfunction when the patients are in a cold environment.^[3]^ Most cases of CAS are complicated with lymphoma, and some other hematological malignancies and cases of CAS with solid tumors have been seldom reported.^[4]^ Surgical resection is always the unique way to achieve curative outcome for the solid tumor patients. For these patients with CAS, the perioperative management should be vital to prevent serious complications caused by CAS. However, few reported literatures are available about the experience of perioperative management for such patients after searching Pubmed and Web of Science. After careful searching for the literatures written in Chinese and English, the authors did not find any reported case of the perioperative management of CAS complicated with gastric cancer. The case reported in this paper could be the first one. The conditions of this patient are very sensitive to the environment, and the exacebations of CAS could lead to serious complications, so the meticulous perioperative management is the prerequisition to avoid such dismal outcome. As this case report could be the first one, it will be of great value to spread our knowledge to provide better perioperative managements for such patients.

## 2 Case report

**Figure 1:**
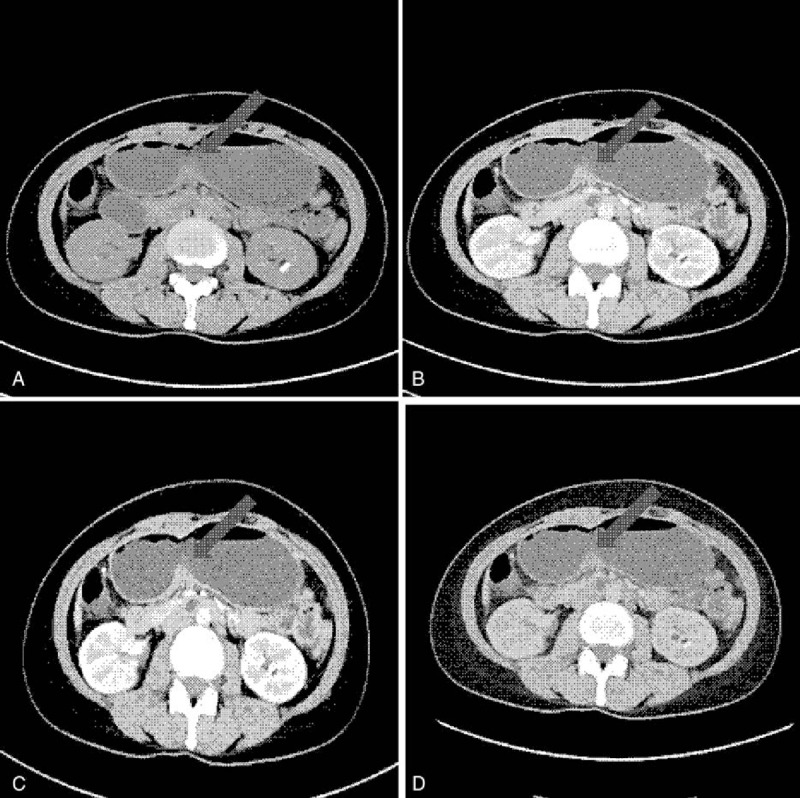
the pictures of intravenous enhanced computed tomography scan of this patient: (A): plain phase; (B): aterial phase; (C): portal vein phase; (D): delayed phase.

Our report did not disclose the patient's privacy. We also did not bring potential harm or any other discomfort to the patient. We gave the consent form to the patient and he signed it. The patient was a 54-year-old man, he underwent a gastroscopy examination because of 3-month diarrhea, and the pathological result demonstrated poorly differentiated adenocarcinoma of the gastric antrum (Fig. 1). The positron emission tomography–computed tomography (PET-CT) indicated that it was a advanced gastric cancer with possible peri-gastric lymph node metastasis. Eight years ago, the patient was diagnosed with CAS. The symptom was a dark or purple discoloration of his fingertips, toes, nose, and ears when exposed to cold ambient temperatures. He was treated with plasmapheresis and immunosuppressants but the treatment outcome was unsatisfactory. After multidisplinary team discussion, 2-month neoadjuvant chemoradiotherapy was performed for him. Four weeks after the neoadjuvant chemoradiotherapy, the patient was admitted to our general surgery department for operation consideration, and he was placed in a pre-warmed ward set at 30°C soon after admission.

The results of preoperative examination were as follows: hemoglobin 109 g/L, alanine and aspartate aminotransferase normal, total/direct bilirubin 25.6/10.4 μmol/L, IgM 5.0 g/L, complement component C3 0.753 g/L, and complement component C4 0.002 g/L. An antiglobulin test showed: Coombs’ (+), IgM (+), C3 (+ + + +), immunoglobulin A (IgA) (−), and immunoglobulin G (IgG) (−). Serum immunofixation electrophoresis showed: IgM-k (+), I (−), and others (−). The cold agglutinin titer was: 37°C no activity, 25°C (room temperature) 1:32, 4°C 1:2048, and D-L antibody test (+).

Two weeks later, he underwent open radical distal gastrectomy and D2 lymph node resection under general anesthesia. The operation was uneventful without blood transfusion. In a stable condition, he returned to the ward with a stomach tube, central venous catheter, jejunostomy tube, abdominal drainage, and urinary catheter. We set the ward temperature at 30°C and offered him a warmed-up infusion. In addition, the patient was fed with enteral nutritionthrough a jejunostomy tube with a warmed feeding pump.

The postoperative period was unenventful without jaundice, hemoglobinuria, livedo reticularis, or acrocyanosis. He was discharged from the hospital 8 days after operation with a jejunostomy tube.

## 3 Experience of perioperative management

### 3.1 Preoperative managements

#### 3.1.1 Psychological nursing

The patient had CAS for 8 years, and the diagnosis of gastric cancer undoubtedly made the condition worse. To relieve his psychological stress, we invited patients who had undergone gastric operations to share their experiences with him. We also discussed the disease with the patient in detail and explained the whole exact plan to him including how to avoid exacerbations of CAS and how to face possible complications caused by CAS. As a result of these psychological interventions, the patient became more confident with the treatment and was able to cooperate actively.

#### 3.1.2 Preoperative preparation

On the morning of the operation, the patient took routine medications for CAS. The temperature of the operating room was adjusted to 29°C–30°C with 50% humidity. Liquids used during surgery were placed in an incubator in the operating room and maintained at 37°C. To reduce waiting time, this operation was scheduled to be first one, and the patient was covered with a hot-air warm blanket when he was transferred from the ward to the operating room.

### 3.2 Intraoperative managements

#### 3.2.1 Temperature monitoring

**Figure 2:**
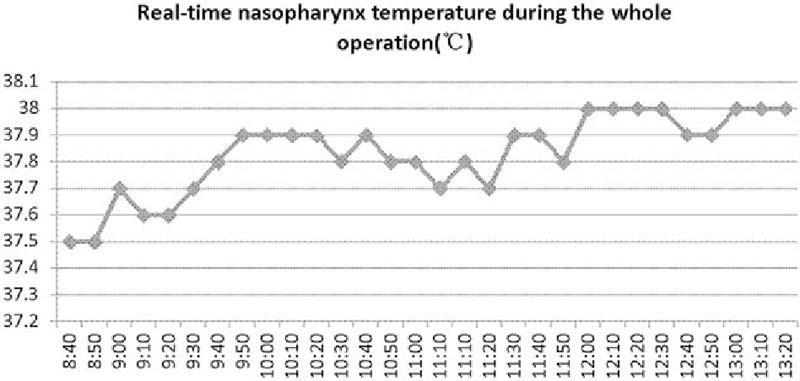
Real-time nasopharynx temperature during the whole operation (°C): the nasopharynx temperature of the patient stably ranged from 37.5°C to 38.0°C during operation.

Temperature was the main focus during the operation. Before surgery, the operating room was warmed by the air-conditioning unit. The patient's upper and lower extremities were carefully covered with pre-warmed blankets. Anesthetic gases and all fluids, including intravenous fluids, irrigation fluids, blood products, and intravenous drugs were warmed and maintained at 37°C–38°C before use. Core and skin temperatures were monitored by nasopharyngeal temperature sensors throughout surgery.^[5]^Figure 2 shows the real-time nasopharyngeal temperatures during the operation.

#### 3.2.2 Cooperation with blood bank

Special blood-warming equipment was on standby in the blood transfusion department. If necessary, erythrocytes could be warmed from 4°C to 30°C within 15 minutes.

### 3.3 Postoperative managements

#### 3.3.1 Heat preservation nursing

Room temperature was set at 28°C–30°C by the air-conditioning unit and the electric heating equipment. The patient was kept warm with a hot-air warm blanket or a cotton quilt.

#### 3.3.2 Vital signs monitoring

Vital signs, especially body temperature, were monitored. We measured the vital signs every 2 hours and recorded the details accurately. Nurses observed the patient's skin color closely during hospitalization. The patient had a fever 2 days after operation, up to 38.5°C. To prevent condensation, the warm water bath was used instead of ice packs to control the fever.

#### 3.3.3 Heated transfusion

To ensure that warm liquid was infused into the body, we set the heating bar to 37°C, and the infusion tube was warmed by the heating bar at 7 cm from the puncture point.

#### 3.3.4 Urine observation

A major complication of CAS is acute renal failure caused by hemolysis. To avoid such a complication, we observed the urine color closely every day. For this patient, the urine test after operation indicated that there was no hemoglobinuria.

#### 3.3.5 Blood specimen collection and transmission

To avoid coagulation of the blood samples, we cooperated closely with the laboratory service. Blood specimens were immediately put into a prepared thermos cup which was filled with warm water at 37°C. The specimens were sent to the laboratory by specially assigned personnel soon after collection.

#### 3.3.6 Enteral nutrition care

After the recovery of his intestinal function, the patient started enteral nutrition. Two days after the operation, 40.2 g Weaver, an amino acid nutrition powder, plus 150 mL water was administered through the jejunostomy tube at a rate of 20 mL/h. Enteral nutrition was increased to the level near body temperature,^[6]^ which was ∼37°C. During pumping, we observed whether the patient experienced bloating or abdominal pain. On the fifth day, 500 mL Weaver and 500 mL of anenteral nutritional suspension, a short-peptide nutrient solution, was administered and the pump rate was increased to 50 mL/h. The patient started to drink a small amount of warm water. Before enteral nutrition is administered, the pipe line must be fixed properly, and to avoid blockage, the jejunostomy tube should be washed with 20–40 mL warm water once or twice before, during, and after the feeding process. Four days after the operation, the jejunostomy tube began to develop resistance. According to our experience of such a situation, a carbonated beverage was used to wash the tube, which led to a marked improvement.

#### 3.3.7 Discharge education

On the day of his discharge, the patient was told to take several important precautions. First, he was instructed to keep his body temperature stable and warm. If the ambient temperature was low or if he felt cold, he should wear gloves, earmuffs, and a scarf. Second, he was advised to take his medication on time. Third, we taught the patient to observe his urine color by himself. If his urine became dark brown, he should see his doctor immediately, as this would indicate hemolysis. Finally, we showed the patient how to care for the jejunostomy tube in details.

## 4 Discussion

When CAS patients undergo major surgery, special assessments and management should be implemented. If the temperature is low in the operating room, agglutination may occur, causing acrocyanosis, Reynaud's phenomenon, purpura, acryl gangrene, or immune complex nephritis.^[7]^ Hemolysis may also occur, resulting in severe anemia, hemoglobinuria, and even renal failure. All these are catastrophic outcomes for both patients and surgeons. There are some reports of patients with CAS undergoing cardiac surgery, which has a major impact on body temperature.^[8]^ We believe this reported case is the first one of gastric surgery on a patient with a long history of CAS.

During perioperative practice, we should assess the patients’ general condition, paying particular attention to appropriate medication, and provide comprehensive and effective instruction and psychological support. All temperature maintenance, in cases like the one described in this paper, should be carried out before the operation, including room temperature setting, infusion temperature warming, and maintenance of blood specimen temperature. Intraoperative management should focus on warming the patient and the fluids. Core and skin temperatures should be monitored throughout operation. To facilitate the recovery of gastrointestinal functions, early enteral nutrition support should be implemented and infusion temperature should be controlled. In addition, before patients are released from hospital, comprehensive instruction and precautions should be given. Most importantly, patients should be instructed always to maintain their body temperature stable and warm with caution of possible serious complications caused by cold ambient temperatures.

In conclusion, perioperative management of CAS patients is a special practice. To avoid the catastrophic occurrence of hemolysis, comprehensive preoperative assessments and systematic body temperature maintenance throughout hospitalization are essential. In this paper, we reported our perioperative management experience of a patient with gastric cancer complicated with CAS. This experience should be valuable to add our knowledge of clinical practice for these patients.
